# Incidental diagnosis of struma ovarii through radioiodine whole-body scanning: incremental role of SPECT/CT

**DOI:** 10.1590/0100-3984.2015.0027

**Published:** 2016

**Authors:** Rômulo Hermeto Bueno do Vale, Heitor Naoki Sado, Débora Lucia Seguro Danilovic, Pulo Schiavom Duarte, Marcelo Tatit Sapienza

**Affiliations:** 1 Instituto do Câncer do Estado de São Paulo Octavio Frias de Oliveira (Icesp), São Paulo, SP, Brazil.

**Figure 1:**
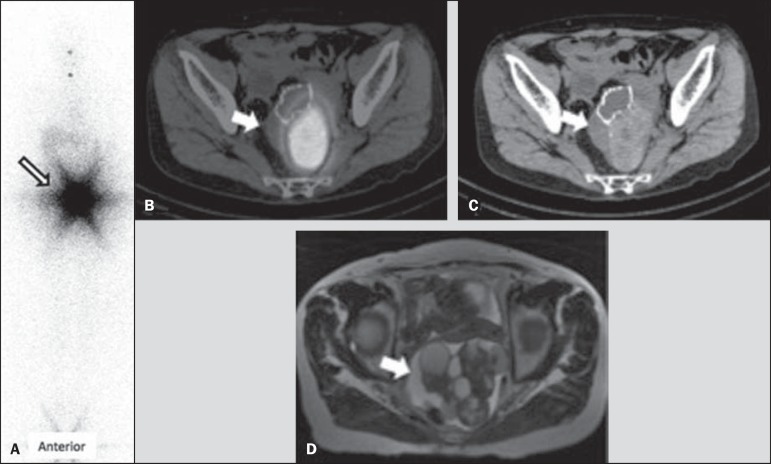
**A:** Anterior view of pre-treatment radioiodine whole-body scan showing an area of intense pelvic uptake (arrow). **B:** Fused SPECT/CT images. **C:** CT image. The arrows indicate a heterogeneous pelvic mass located posterior to the uterus. **D:** Axial T2-weighted magnetic resonance imaging scan. The arrow indicates a lobulated multicystic pelvic mass with a solid component.

Dear Editor,

A 76-year-old woman with papillary thyroid cancer (staging: pT3pN1pMx) was referred for radioiodine (I-131) therapy after total thyroidectomy. The thyroglobulin titer was elevated (190 ng/mL) and thyroid stimulating hormone (TSH) levels remained suppressed despite thyroxine withdrawal. A radioiodine whole-body scan (WBS) evinced an area of intense pelvic uptake (Figure 1A), which corresponded to a heterogeneous pelvic mass posteriorly to uterus on fused single-photon emission computed tomography/computed tomography (SPECT/CT) images (Figure 1B). The SPECT/CT findings suggested a diagnosis of struma ovarii. Complementary pelvic magnetic resonance imaging depicted a lobulated multicystic pelvic mass with a solid component, probably originating from the left ovary (Figures 1C and 1D). Total hysterectomy was performed, revealing a mature teratoma with thyroid tissue (struma ovarii). Five months after surgical resection, the patient was treated with 7400 MBq (200 mCi) of I-131. A post-treatment radioiodine WBS evinced a cervical thyroidal remnant and a focal area of increased radioiodine uptake in the proximal diaphysis of the left femur, with no matching alteration on CT images (not shown). The TSH-stimulated thyroglobulin titer was 2.4 ng/mL (normal value, < 35.0 ng/mL). At month 3 of clinical follow-up, the patient had not presented any symptoms concerning the left lower limb and the thyroglobulin titer remained at a normal range.

Images obtained in a radioiodine WBS are noisy and have low spatial resolution. It is therefore often difficult to do proper anatomical localization, basically due to the high-energy characteristics of radioiodine^(1)^. At our institution, we often resort to SPECT/CT to further evaluate cases with inconclusive findings on standard planar scintigraphic images. The role of SPECT/CT for the evaluation of patients with well-differentiated thyroid carcinoma has not yet been established, although a few studies have demonstrated its superiority in the localization and identification of metastatic lesions^(2,3)^. In the case presented here, we believe that SPECT/CT played an important role in the correct anatomical location of the pelvic focal uptake, as well as improving the differential diagnosis by adding tomographic features that supported the hypothesis of struma ovarii^(4,5)^.

Surgical resection is the mainstay of struma ovarii treatment^(6,7)^, and it is recommended that patients be closely monitored through sequential thyroglobulin measurements, along with radioiodine WBS if recurrence is suspected, during follow-up. For malignant struma ovarii, a longer follow-up period is recommended, usually more than 10 years. Adjuvant I-131 therapy (after total thyroidectomy) might be considered in some patients^(6)^ . In our case, post-treatment radioiodine WBS evinced a focal area of radioiodine uptake in the proximal left femur, probably due to benign and nonspecific etiology rather than metastatic disease, given that there were no matching anatomic alterations or symptoms. Subsequent follow-up will be needed in order to confirm that impression. In conclusion, fused SPECT/CT images played an important role in the differential diagnosis of a benign pelvic mass incidentally detected in a pre-treatment radioiodine WBS in a patient with papillary thyroid carcinoma.
